# UV-B Radiation Enhances *Epimedium brevicornu* Maxim. Quality by Improving the Leaf Structure and Increasing the Icaritin Content

**DOI:** 10.3390/plants13131720

**Published:** 2024-06-21

**Authors:** Pengshu Li, Qiuyan Xiang, Yue Wang, Xuehui Dong

**Affiliations:** 1 College of Agronomy and Biotechnology, Sanya Institute of College of China Agricultural University, Sanya 610101, China; 2 College of Agronomy and Biotechnology, China Agricultural University, Beijing 100193, China; b20213010057@cau.edu.cn; 3 Shandong Academy of Agricultural Sciences, Jinan 250100, China

**Keywords:** *Epimedium brevicornu* Maxim., UV-B radiation, metabolomics

## Abstract

*Epimedium brevicornu* Maxim. is a herbal plant with various therapeutic effects, and its aboveground tissues contain flavonol compounds such as icaritin that can be used to produce new drugs for the treatment of advanced liver cancer. Previous studies have shown that ultraviolet-B (UV-B, 280–315 nm) stress can increase the levels of flavonoid substances in plants. In the current study, we observed the microstructure of *E. brevicornu* leaves after 0, 5, 10, 15, and 20 d of UV-B radiation (60 μw·cm^−2^) and quality formation mechanism of *E. brevicornu* leaves after 0, 10, and 20 d of UV-B radiation by LC‒ESI‒MS/MS. The contents of flavonols such as icariside I, wushanicaritin, icaritin, and kumatakenin were significantly upregulated after 10 d of radiation. The results indicated that UV-B radiation for 10 d inhibited the morphological development of *E. brevicornu* but increased the content of active medicinal components, providing a positive strategy for epimedium quality improvement.

## 1. Introduction

UV-B radiation induces the rapid accumulation of flavonoids in the leaf epidermis [1]. The removal of reactive oxygen species by the functional group isomerism and the protection of reactive groups by the glycosidic glycosylation in flavonoids make them the main UV-radiation-resistant substances in plant cells [2,3,4]. *Epimedium brevicornu* Maxim. is a perennial persistent C3 plant of the genus *Epimedium* Linn. in the family *Berberaceae* with approximately 300 detected flavonoids. The flavonol with the highest content in the leaves is icariin, which exerts pharmacological effects such as regulating T-cell homeostasis to enhance immune function, reducing highly metastatic human lung giant-cell carcinoma cell motility to reverse malignant tumour cells, fighting liver toxins to improve cardiovascular and cerebrovascular function and promoting estrogen biosynthesis in vivo [5,6,7,8]. Icariin, combined with epimedin A, epimedin B, and epimedin C serves as an index for evaluating the quality of herbs belonging to the genus *Epimedium*. Previous studies have shown that light quality [9,10] and intensity [11,12] could influence the active component content of *Epimedium*; however, the effects of UV-B radiation on *E. brevicornu* are largely unknown. Several studies on the secondary metabolites of other species under UV-B stress have been performed in the past years. Liu et al. used GS/MS untargeted metabolomics combined with LC/MS targeted metabolomics techniques to construct a network of interactions between primary and phenolic metabolism and showed that phenolics with C6C3 (coumarins and lignans) and C6C3C6 (flavonoids) carbon skeletons accumulated to significantly higher levels in *Astragalus membranaceus* Bge. var. *mongholicus* Hsiao under UV-B and drought stress, whereas relatively lower accumulation of alkanols was observed, indicating that this species shows stronger stress tolerance than *Astragalus membranaceus* (Fisch.) Bge. [13]. A metabolomic analysis of *Glycyrrhiza uralensis* Fisch leaves irradiated with a low dose (2.4 μw· cm^−2^) of UV-B by HPLC-ESI-TOF-MS/MS showed that the contents of flavonoids and phenolics first showed an increasing trend; however, with an increase in the radiation time, the plant cells became damaged, and the content of substances gradually decreased [14]. In addition, other studies have investigated the UV-B-induced changes in the content of secondary metabolites in medicinal plants. For example, the exposure of *Dendrobium officinale* Kimura et Migo to UV-B stress induces the biosynthesis of alkaloids, polysaccharides, and flavonoids, inducing many key biosynthetic pathways, such as glycolysis, starch and sucrose metabolism, and fructose and mannose metabolism [15]. The anthocyanins, flavonoids, and phenolic contents of saffron (*Crocus sativus* L.) increase after exposure to UV-B stress [16]. In *Hypericum perforatum*, 0.430 μg/g DW hypericin is detected after 60 min of UV-B radiation, and this value was eight times the original yield [17].

Upon exposure to UV-B stress, plants stimulate stress defense response mechanisms in the body, and the most direct response to this stress is the effect on plant growth and morphological development [18]. Significant thickening of palisade cells and a significant increase in the palisade cell–spongy mesophyll ratio have been observed in mango (*Mangifera indica* L.) leaves after continuous treatment with 96 kJ· m^−2^· d^−1^ UV-B radiation for 5 months [19]. In rice (*Oryza sativa* L.), leaves show sunken stomata on both sides of the main veins after 21 d of 18.6 kJ· m^−2^ · d^−1^ UV-B radiation [20]. In *Rosa roxburghii* (*R. roxburghii* Tratt.), subsidiary cells of the leaves are subjected to destruction after 60 d of 12.4 kJ· m^−2^ · d^−1^ UV-B radiation [21]. The effects of UV-B radiation on the leaf ultrastructure are mainly damage to the membrane system of chloroplasts, enlargement of granum thylakoids, and a loose and disordered arrangement in the lamella of the granum and stroma [22,23]. In addition, increases in the numbers of osmiophilic granules and degradation of starch granules were found in the chloroplasts of grape (*Vitis vinifera* L.) [24] leaves after 18 d of 0.3 w·m^−2^ UV-B radiation and mango [25] leaves after 16 months of 5.0 kJ· m^−2^ · d^−1^ UV-B radiation.

*E. brevicornu* is considered a traditional Chinese medicine with growing commercial value. To investigate the effectiveness of UV-B radiation in enhancing the main medicinal components in Epimedium, we observed the leaf area, microstructure, and chloroplast ultrastructure of *E. brevicornu* after exposure to radiation for different times and explored the relationship between UV-B radiation and the formation of secondary metabolites by widely targeted metabolomics to provide a reference for the selection and quality control of *Epimedium* and thus guidelines for the good agricultural practice (GAP) of this medicinal plant.

## 2. Results

### 2.1. Effects of UV-B Radiation on the Tissue Structure and Secondary Metabolites of E. brevicornu Leaves

**Figure A1:**
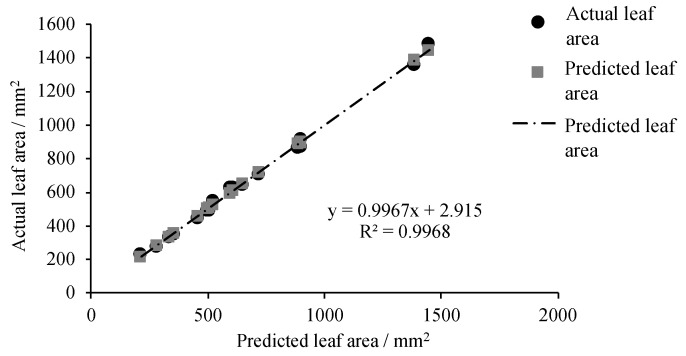
Correlation analysis between the predicted leaf area and actual leaf area.

The fitted function Y = 0.672X3 + 1.723X4 − 56.946 for the correlation factors with leaf length + leaf width and leaf length × leaf width was selected as the best model for estimating the *E. brevicornu* leaf area with a maximum multiple correlation coefficient value of 0.997 and a minimum mean absolute error of 16.415 (Table A1). The R^2^ value of the regression equation between the predicted leaf area and the actual leaf area is 0.9968 (Figure A1), indicating that the fitting function is available.

**Figure 1:**
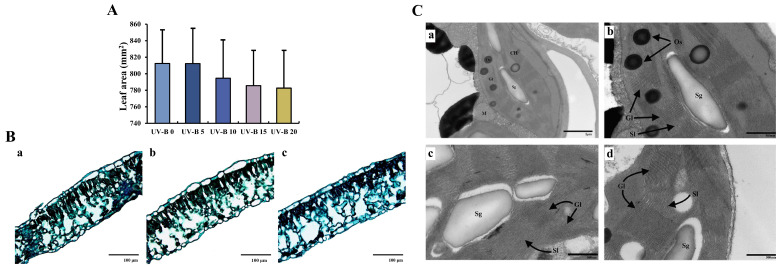
Effect of UV-B radiation on *E. brevicornu* leaves: (**A**) Changes in the *E. brevicornu* leaf area under UV-B radiation for different numbers of days. (**B**) Microstructure of *E. brevicornu* leaves under UV-B radiation for different numbers of days. (**a**–**c**) show the microstructure of *E. brevicornu* leaves at 0, 10, and 20 d, respectively. (**C**) Ultrastructure of *E. brevicornu* chloroplasts under UV-B radiation for different numbers of days. (**a**–**d**) show the chloroplast ultrastructure after 0, 10, and 20 d, respectively. CH: chloroplast; M: mitochondrion; Sg: starch granule; Gl: granum lamella; Sl: stroma lamella; Os: osmiophilic granule.

The fitting function was used to calculate the *E. brevicornu* leaf area after UV-B radiation for different days (Figure 1A). The growth and development of epimedium leaves were inhibited with increases in the radiation days, and the leaf area gradually decreased after 10 d of radiation.

Based on the data in Figure 1B and Table 1, the thickness of the upper epidermis and palisade cells was significantly decreased after 20 d of radiation compared with 0 d. The thickness of the upper epidermis decreased from 13.26 ± 7.58 to 7.83 ± 1.38 μm, and the thickness of palisade cells decreased from 35.00 ± 8.85 to 22.83 ± 3.05 μm. Due to the significant decrease in the palisade cell thickness, the palisade cell–spongy mesophyll ratio decreased significantly from 595.60 ± 97.67 to 381.08 ± 55.59 μm after 15 d of radiation. No significant change in the thickness of the spongy mesophyll and lower epidermis was observed.

The analysis of the ultrastructure of *E. brevicornu* leaves (Figure 1C) showed that the central large vacuoles occupied most of the mesophyll cells without UV-B radiation. Chloroplasts and mitochondria were located at the edge of the cell. Chloroplasts were shuttle-shaped and closely connected with the cell wall, with a complete double-layer membrane structure. Stroma lamellae were stacked neatly and orderly, and granum lamellae were closely attached. After 10 d of UV-B radiation, the number of starch granules in chloroplasts was increased, the granum lamella structure began to separate, and the gaps increased. After 20 d of UV-B radiation, the stroma lamella and granum lamella were distorted and arranged in a disorderly manner, and the number of starch granules was decreased compared with that observed after 10 d of radiation.

The content of the six flavonols in *E. brevicornu* was determined by HPLC, and the R^2^ values of the standard regression equation were higher than 0.9990 in the linear range (Table A2).

**Figure 2:**
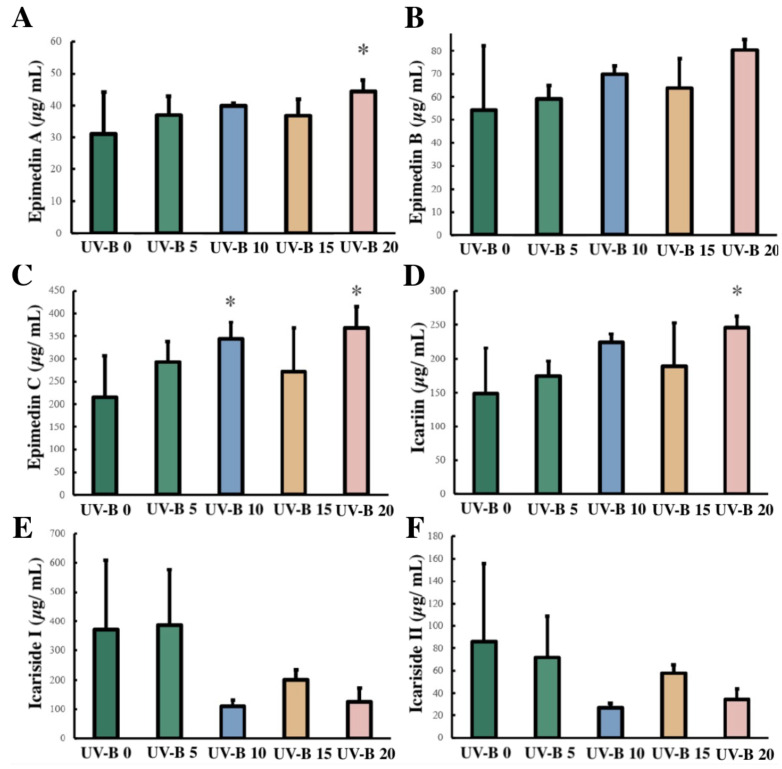
Changes in the contents of six secondary metabolites under UV-B radiation for different times. (**A**) Epimedin A content, (**B**) Epimedin B content, (**C**) Epimedin C content, (**D**) Icariin content, (**E**) Icariside I content, (**F**) Icariside II content. * *p* < 0.05.

As shown in Figure 2, an overall increasing trend of epimedin A, epimedin B, epimedin C, and icariin content was found in the leaves with increases in the number of days of UV-B radiation. The contents of epimedin A and icariin increased significantly from initial values of 31.00 and 148.88 μg/mL to 44.39 and 235.99 μg/mL after 20 d of radiation, respectively. The epimedin C content increased from an initial value of 215.97 to 340.358 μg/mL after 10 d of radiation. The contents of icariside I and II decreased with increases in the number of days of radiation, but these decreases were not significant.

### 2.2. Analysis of Metabolites during Radiation Exposure

**Figure 3:**
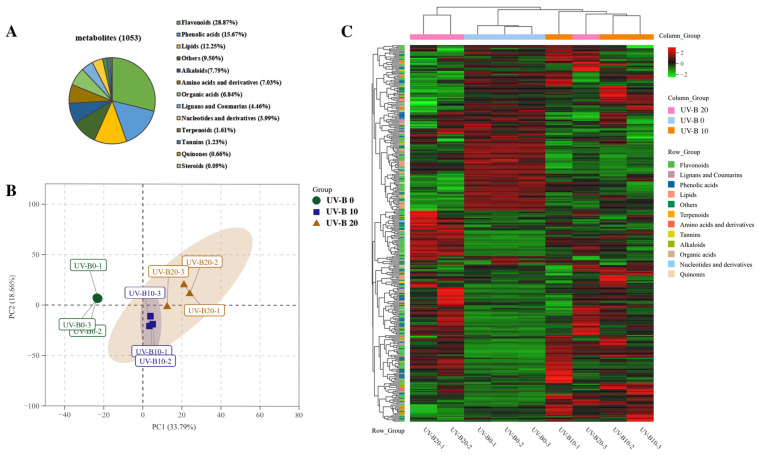
Metabolite analysis of *E. brevicornu*: (**A**) Total detected metabolites. (**B**) PCA plot of *E. brevicornu* at three time points; 0, 10, and 20 d refer to 0, 10 and 20 days of UV-B radiation. (**C**) HCA heatmap of *E. brevicornu* metabolites at three time points.

According to the contents of the six flavonols (stipulated in Chinese Pharmacopoeia) and the morphological structures of *E. brevicornu*, there are only significant changes after an irradiation period of 10 d and 20 d comparing with the control. So, leaves were picked at three time points (UV-B 0, 10, and 20 d) to study the metabolic changes during exposure to radiation. A total of 1053 metabolites were detected in the metabolomic analysis. The metabolites were divided into thirteen groups (Figure 3A): flavonoids, phenolic acids, lipids, alkaloids, amino acids, organic acids, lignans and coumarins, nucleotides, terpenoids, tannins, quinones, steroids, and others. Notably, the 304 flavonoids contained 38.49% flavonols (Table A3), which were the main medicinal ingredients in *E. brevicornu*.

The principal component analysis (PCA) plot showed that the 1053 metabolites could be obviously divided into three groups, each of which was associated with a different treatment (Figure 3B). In line with the PCA results, principal component 1 (PC1) explained 33.79% of the total metabolite characteristics. A significant separation could be found between the UV-B 0 treatment group and the other two groups, which indicated that the UV-B treatment induced a significant change in metabolites, consistent with the detected changes in physiological indicators such as the leaf area, leaf microstructure, and chloroplast ultrastructure.

The differences in the accumulation patterns of metabolites after radiation for different numbers of days could be analyzed by hierarchical clustering analysis (HCA) (Figure 3C). The results of the analysis showed significant differences in the metabolites between different groups, and these metabolites were divided into two clusters in total. The 156 metabolites in cluster 1 included flavonoids and lipids, and their content decreased with increases in the number of radiation days. The 197 metabolites in cluster 2 included flavonoids, alkaloids, and phenolic acids, and their content increased with increases in the number of radiation days. Different biological replicates were clustered together, indicating good homogeneity between the biological replicates and high reliability of the data.

### 2.3. Analysis of Differential Metabolites

**Figure A2:**
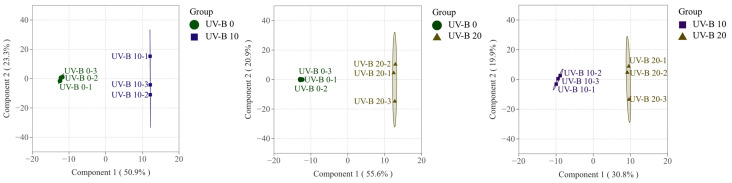
OPLS-DA analysis.

Orthogonal partial least squares discriminant analysis (OPLS-DA) (FC ≥ 2 and VIP ≥ 1) was applied to detect the differential metabolites (DMs) among different treatments by removing effects that were not relevant to the study. As shown, significant separation was found among the different comparison treatment groups (Figure A2).

**Figure 4:**
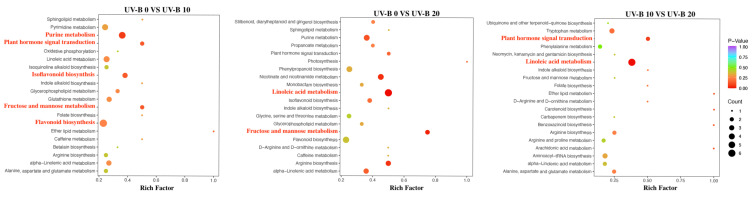
KEGG enrichment analysis.

The KEGG analysis of the DMs between each pair of treatments is shown in Figure 4. The DMs obtained from the UV-B 0 vs. UV-B 10 comparison participated in the metabolic pathways of isoflavonoid biosynthesis and flavonoid biosynthesis, suggesting that flavonoid metabolism might play a critical role in *E. brevicornu* leaves after 10 d of UV-B radiation. In addition, the plant hormone signal transduction pathway and linoleic acid metabolism pathway were also enriched in the DMs identified from the UV-B 0 vs. UV-B 20 and UV-B 10 vs. UV-B 20 comparisons, respectively.

**Figure 5:**
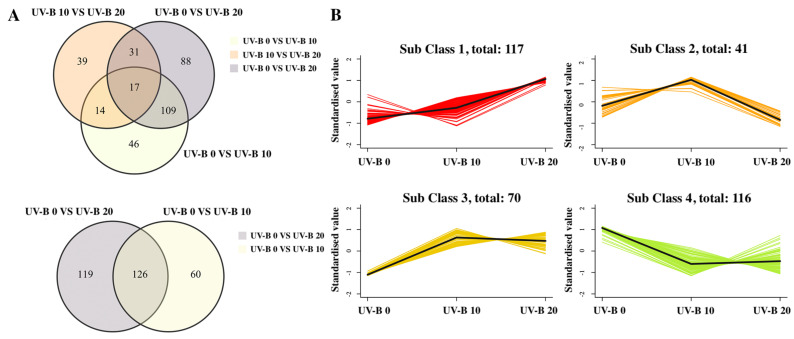
Differential metabolite analysis: (**A**) Venn plot. The numbers represent the identified DMs. (**B**) K-means clustering algorithm analysis of the DMs.

A Venn plot was constructed using the DMs identified from the UV-B 0 vs. UV-B 10, UV-B 0 vs. UV-B 20, and UV-B 10 vs. UV-B 20 comparisons (Figure 5A). To study the crucial metabolites under UV-B radiation, 17 and 126 overlapping DMs were screened. Furthermore, we found four different trends for these DMs among the three treatments (Figure 5B). For instance, 187 metabolites showed a higher content in the UV-B 10 and UV-B 20 groups than in the UV-B 0 group. A total of 116 metabolites showed higher accumulation in the UV-B 0 group than in the UV-B 20 group. Among the 17 overlapping DMs, subclass 1 contained phenolic acids, lignans, and alkaloids, and subclass 4 included flavonoids such as baohuoside II, baohuoside III, ikarisoside F and 7-O-methylnaringenin (Table A4).

### 2.4. Metabolites Related to *E. brevicornu* Quality Traits in the Three Treatment Groups

Flavonols are the main medicinal components of *E. brevicornu*. In flavonoid metabolism, some flavonols, such as icaritin, wushanicaritin, and icariside I, showed increases in accumulation with the extension of the UV-B radiation time, whereas others, such as baohuoside II, baohuoside III, and ikarisoside F, showed decreases (Table 2 and Table 3).

**Figure 6:**
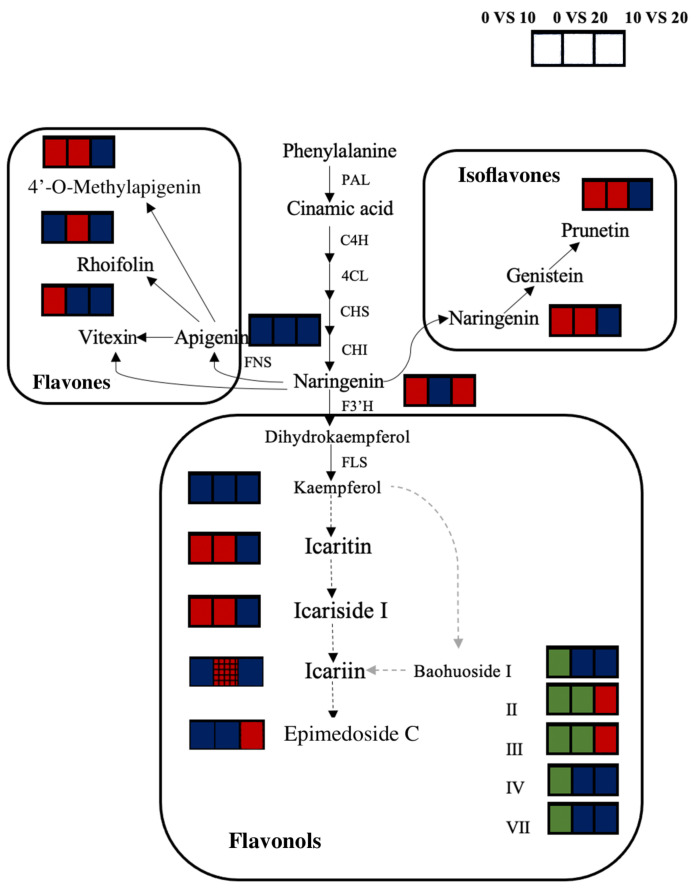
Analysis of key regulatory pathways of flavonoid biosynthesis identified in the UV-B radiation experiments. The black solid lines represent annotated pathways in the KEGG database; the black and grey dashed lines represent pathways proposed by Yang [26] and Feng [27]. The three boxes from left to right correspond to UV-B 0 vs. UV-B 10, UV-B 0 vs. UV-B 20 and UV-B 10 vs. UV-B 20, respectively. Significantly increased metabolites are filled with red; significantly decreased metabolites are filled with green; metabolites with no significant change are filled with blue; the red black grid squares represent an increasing metabolite content determined by HPLC (Figure 2). PAL, phenylalanine ammonia-lyase; C4H, cinnamate-4-hydroxylase; 4CL, 4-coumarate:CoA ligase; CHS, chalcone synthase; CHI, chalcone isomerase; F3’H, flavanone 3-hydroxylase; FLS, flavonol synthase; FNS, flavone synthase.

The changes in the content of each metabolite in the flavonoid biosynthesis pathway (map00941) are shown in Figure 6. In the isoflavonoid biosynthesis pathway (map00943), an increased naringenin content in UV-B-irradiated *E. brevicornu* leaves led to a significant increase in the content of prunetin synthesized from genistein. In the flavone and flavonol biosynthetic pathway (map00944), apigenin alone synthesized 4’-O-methylapigenin, and the content of rhoifolin showed a significant increase after radiation. The level of vitexin, which is synthesized by both apigenin and naringenin, increased significantly after 10 d of radiation. The metabolism of flavonols in the KEGG database was only updated to kaempferol, and the biosynthesis pathways of several flavonol metabolites shown by the dashed lines in Figure 6 were inferred from the relevant literature and chemical structure. Among these, icaritin and icariside I, which are synthesized from kaempferol, were significantly increased after radiation, whereas the contents of baohuoside II, baohuoside III, and epimedoside C were significantly decreased after 10 d of radiation.

## 3. Discussion

### 3.1. Effect of UV-B Radiation on the Leaf Structure

UV-B radiation can induce morphological changes in plants, with the effects being time- and dose-dependent [28]. Plants subjected to UV-B stress exhibit a decrease in the leaf area of newborn leaves [29,30] and leaf thickening [31,32]. The *E. brevicornu* leaf area decreased after 10 d of UV-B radiation, whereas the HPLC results showed an increasing trend for the epimedin A, epimedin B, epimedin C, and icariin contents in the leaves. Previous studies have shown that the flavonoid content in the genus Epimedium is significantly correlated with leaf area (*p* < 0.05), presumably because leaves with smaller leaf areas usually need more flavonoids to protect the leaves from damage because they are less leathery [33].

Starch granules in the chloroplast ultrastructure exist in granular form as temporary storage locations for photosynthesis products. The number of starch granules changes significantly under different light qualities and light intensities. Cucumber (*Cucumis sativus* L.) leaves under blue light radiation significantly reduce the outwards output of photosynthesis products, increasing the number of starch granules in the chloroplast and the feedback inhibition of photosynthesis, which reduces the photosynthesis capacity of the leaves [34]. Tomato (*Lycopersicon esculentum* L.), *Trollius chinensis* (*T. chinensis* Bunge), and peach (*Amygdalus persica* L.) leaves under shade and dim light treatments show significant decreases in the number of starch granules due to decreases in the total photosynthesis products [35,36,37]. Studies have also shown that abiotic stress conditions such as high ammonia, nitrogen, and magnesium caused significant increases in the number of starch granules in the leaves of several plants compared with those found with the control treatment [38,39,40,41]. Therefore, the number of starch granules in chloroplasts increased significantly after 10 d of UV-B radiation, presumably due to the stress effect of UV-B radiation on photosynthesis in *E. brevicornu* leaves, which reduced the output of photosynthesis products and thus increased the number of starch granules.

### 3.2. Effect of UV-B Radiation on Secondary Metabolites of E. brevicornu

Plant secondary metabolites are usually classified into three major groups: phenolic compounds (flavonoids, simple phenols, and quinones), terpenoids, and nitrogen/sulfur-containing organic compounds (alkaloids and amines). UV radiation has long been considered an effective exciter to promote the biosynthesis of various plant secondary metabolites, and endogenous NO induces the accumulation of secondary metabolites synthesized as an early stress response, whereas flavonoids are activated as secondary antioxidant systems [42,43].

Flavonoids are involved in a variety of physiologically and biochemically active responses in plants, including pathogenesis and symbiotic relationships, self-protection under UV radiation, pollen development, seed germination, endogenous regulation of polar transport of auxin, and regulation of the cell cycle in higher plants [44,45,46]. Flavonoids share the same flavonoid parent nucleus, and glycosyltransferases play a controlling role in the interconversion of metabolites because they are more sensitive to the environment and a small amount of stimulation can affect their gene expression. Assays of the response of buckwheat to UV-B stress found that the expression of key enzyme-encoding genes of the shikimate-phenylpropanoid metabolic pathway increased significantly and the feedback pressure of the pentose phosphate (PPP) pathway decreased significantly, and these effects were responsible for the significant increases in the flavonoid contents of puerarin, naringin, quercetin, rutin, and kaempferol in the plant [47]. Fewer studies have investigated the interconversion of flavonols in *Epimedium*. Sun et al. found that heating decreased the epimedin A, B, and C contents of the Epimedium cut crude drug and increased the contents of icariin and baohuoside I, proving that icariin was not entirely converted from the other three flavonoids (epimedin A, B, and C) but rather produced through a complex conversion process involving multiple components in *Epimedium* [48]. Huang et al. predicted the expression pattern of the flavonoid pathway and flavonoid-related enzyme-encoding genes in Epimedium leaves and found that the flux of metabolites in the flavonoid metabolic pathway could go directly from the anthocyanin branch to the flavonol branch after enhancing the expression of EsFLS genes [49,50].

The common DMs obtained from the Venn analysis were classified into six categories in addition to flavonoids, and among these, the metabolites whose content increased after UV-B radiation were phenolic acids, lignans and coumarins, tannins, alkaloids, and organic acids, whereas those whose content decreases were lipids. Studies have shown that substances such as phenolics and alkaloids accumulate in small amounts under UV-B radiation to reduce radiation damage [51]. Phenolic acids are strongly oxidizing due to the phagocytosis of free radicals induced by hydroxy-substituted reactions [52]. Wang et al., radiated 84 K poplar plantlets for 8–16 h using UV-B at an intensity of 3 W·m^−2^ and found significant accumulation of six phenolic acids by UPLC/MS-MS [53]. The alkalinity of alkaloids is derived from nitrogen-containing heterocyclic structures, and although UV-B radiation induces their synthesis, the content varies significantly depending on the plant species, age, organ, and experimental method [54,55,56].

The KEGG database annotation and pathway metabolic analysis performed in the present study showed that the DMs identified from the UV-B 0 vs. UV-B 20 comparison were mainly enriched in the linoleic acid metabolism, arginine biosynthesis, and nicotinate and nicotinamide metabolism pathways. Linoleate and linolenic acid are the major fatty acids in plant membranes and can be metabolized via the octadecanoic acid metabolic pathway to produce hydroxy-phospholipids [57]. The content of unsaturated fatty acids such as lysoPC 18:1(2n isomer), 2-*α*-linolenoyl-glycerol-1,3-di-O-glucoside, and (9Z,12Z)-(7S,8S)-dihydroxyoctadeca-9,12-dienoic acid was significantly decreased in the UV-B 0 group compared with the UV-B 10 group and in the UV-B 0 group compared with the UV-B 20 group (Table A4), whereas the saturated fatty acid index was proportional to the membrane fluidity. UV-B stress altered the ratios of fatty acid fractions, which may be responsible for the observed decrease in the fluidity and integrity of chloroplast membranes, in agreement with the findings reported by Murphy [58]. Abiotic stress has significant effects on the biosynthesis or degradation of certain amino acids [59]. The isoleucine content was significantly increased in the shared DMs of *E. brevicornu* after UV-B radiation. Studies have shown that branched chain amino acids (BCAAs), namely, valine, leucine, and isoleucine, as well as other amino acids that share a synthetic pathway with BCAAs, including lysine, threonine, and methionine, usually accumulate under abiotic stress conditions [60]. The decomposition products of BCAAs directly enter the tricarboxylic acid (TCA) cycle, serve as respiratory substrates under short-day and cold stress conditions and are activated for synthesis under drought stress conditions [61,62,63]. Previous studies have shown that polyamines are also resistant to adversity and increase significantly under exposure to abiotic stresses such as salt, drought, and low temperature [64,65,66,67,68].

## 4. Materials and Methods

### 4.1. Plant Materials

Wild *E. brevicornu* (identified by the researcher Li Xian-en of the Institute of Medicinal Plants Development) plants were transplanted from Zhuoni County (Gansu, China) to Haidian district (Beijing Province, N 40°01′, E 116°16′ H 50 m) in 2016. Five years old plants were used for the research. The degree of photosynthetic active radiation (PAR) and UV-B photon flux density (PFD) in the greenhouse were measured before the experiment. A metal frame (400 cm × 180 cm × 110 cm) was constructed to avoid damage to the surrounding plants and take shelter from the rain; UV-B was isolated with a plastic film on the outside to create a fully closed “shed”. Plants with full and uniform growth traits were selected, 40 plants per treatment, and three replicates were performed. The UV-B radiation intensity was set to 60 μw·cm^−2^ by adjusting the number and height of the lamps (wavelength of 308–310 nm, Beijing Photoelectric Instrument Factory). Four hours (12:00–16:00) of radiation were applied daily for 0, 5, 10, 15, and 20 d. Normal moisture management was applied during this period.

### 4.2. Measurement of the Leaf Length, Width and Area

Because the developmental statuses of a specific leaf needed to be tracked continuously, a total of 90 naturally growing *E. brevicornu* leaves were selected. Sixty of these plants were used to establish a binary regression equation for estimating the leaf area using four indicators, namely, the leaf length, leaf width, leaf length + leaf width, and leaf length × leaf width, as correlation factors. The other 30 plants were used to test the accuracy of the fitted equation. Before UV-B radiation treatment, fifteen leaves of *E. brevicornu* were randomly labeled with three replications. The length and width of the labeled leaves along the main veins and widest part of the leaves were measured by vernier caliper after radiation for five different durations, and we calculated the leaf area according to the fitted equation.

### 4.3. Observation of the Leaf Microstructure

Paraffin sectioning was used for observation of the microstructure of fresh leaves [69]. Ten to fifteen leaves from each treatment group were immersed in a formalin acetate alcohol (FAA) mixture as a fixative and vacuumed for 6–8 min. The samples were dehydrated in a gradient of ethanol and xylene, embedded in wax, and then sliced with a Leica RM2256 into 8 μm serial slices. The slices were then dewaxed in a gradient of xylenol and ethanol and stained with Safranin O-Fast Green. The sections were sealed with Canadian gum and then observed and photographed under an Olympus microscope.

The thickness of the upper epidermis, lower epidermis, palisade cell, and spongy mesophyll were observed and measured under a microscope (OLYMPUS, BX51) at 40 × 10 magnification, and the palisade cell–spongy mesophyll ratio was calculated.

### 4.4. Observation of the Chloroplast Ultrastructure

Five leaves from each treatment group were taken from a 0.2 × 0.2 cm section along both sides of the main veins. The samples were then immersed in Gluta fixative (2.5%, specific for electron microscopy) and vacuumed for 10 min. The sections were rinsed for 2 h using 0.1 M phosphate buffer (pH = 7.0), imbued with acetone, embedded in Epon-812 epoxy resin, polymerized, sectioned, and stained with a mass fraction of 1% uranyl acetate dihydrate. The stained sections were then observed using a transmission electron microscope (Hitachi HT7800, Hitachi Inc., Tokyo, Japan).

### 4.5. Sample Extraction and HPLC Conditions

Sample preparation. Fully developed leaves were collected after 0, 5, 10, 15, and 20 d of radiation. The leaves were dried at 50 °C and milled at 2000 rpm, for 5 cycles. The powder was filtered through a 60-mesh sieve. Subsequently, 0.2 g of powder was weighed precisely and extracted with 20 mL of 70% ethanol aqueous solution. The solution together with the container were weighed, a 1 h’s ultrasonic extraction was performed at room temperature, then weighed again and made up to the weight with 70% ethanol. The test solutions were filtered through 0.22 μm membranes.

Preparation of standard solutions. Reference solutions (2.0 mg/mL) of epimedin A, epimedin B, epimedin C, icariin, icariside I, and icariside II (Chengdu Push Bio-technology Corporation, Chengdu, China) were prepared with methanol in a brown volumetric flask.

The analytical conditions were as follows: HPLC system, Waters ACQUITY ARC System with Waters 2489 UV/Vis Detector (Waters Corporation, Milford, MA, USA); column, Agilent ZORBAX SB-C18 (250 mm × 4.6 mm, 5 μm); solvent system, acetonitrile:water (0.3% formic acid); gradient program, 25:75 *V*/*V* at 0–33 min, 45:55 *V*/*V* at 35 min, 45:55 *V*/*V* at 45 min; temperature, 25 °C; flow rate, 1 mL/min; detection wavelength, 272 nm; and injection volume, 20 μL.

### 4.6. Widely Targeted Metabolomics

Metabolite extraction. Leaves were collected after 0, 10, and 20 d of treatment, freeze-dried using a vacuum freeze-dryer (Scientz-100F, Scientz, Ningbo, China), and then crushed using a mixer mill (MM 400, Retsch) for 1.5 min at 30 Hz. One hundred milligrams of the powder were weighed and dissolved in 1.2 mL of 70% methanol. The dissolved samples were maintained in a refrigerator at 4 °C overnight and vortexed 6 times during this period. After centrifugation at 12,000 rpm for 10 min, the supernatant was extracted and filtered through a 0.22 μm pore-size microporous membrane before UPLC‒MS/MS analysis.

UPLC conditions and ESI-Q TRAP-MS/MS. The sample extracts were analysed using a UPLC‒ESI‒MS/MS system (UPLC, SHIMADZU Nexera X2 system; MS, Applied Biosystem 4500 QTRAP, Waltham, MA, USA). The analytical conditions for UPLC were as follows: column, Agilent SB-C18 (2.1 mm × 100 mm, 1.8 μm); solvent system, water (0.1% formic acid):acetonitrile (0.1% formic acid); gradient program, 95:5 *V*/*V* at 0.0 min, 5:95 *V*/*V* at 9.0 min, 5:95 *V*/*V* at 10.0 min, 95:5 *V*/*V* at 11.1 min, 95:5 *V*/*V* at 14.0 min; flow rate, 0.35 mL/min; temperature, 40 °C; and injection volume, 4 μL. The effluent was alternately connected to an ESI-triple quadrupole-linear ion trap (Q TRAP)-MS.

Linear ion trap (LIT) and triple quadrupole (QQQ) scans were acquired on a triple quadrupole-linear ion trap mass spectrometer (Q TRAP) in an AB 4500 Q TRAP UPLC/MS/MS system equipped with an ESI Turbo Ion-Spray interface operating in the positive and negative ion mode and controlled by Analyst 1.6.3 software (AB Sciex, Framingham, MA, USA). The ESI source operation parameters were as follows: ion source, turbo spray; source temperature, 550 °C; ion spray voltage (IS), 5500 V (positive ion mode)/−4500 V (negative ion mode); ion source gas I (GSI), gas II (GSII), and curtain gas (CUR), 50.0, 60.0, and 25.0 psi, respectively; and collision activated dissociation (CAD), high. Instrument tuning and mass calibration were performed in the QQQ and LIT modes with 10 and 100 μM/L polypropylene glycol solutions, respectively. QQQ scans were acquired as MRM experiments with the collision gas (nitrogen) set to medium. Individual MRM transitions of DP and CE were performed with further DP and CE optimization. Depending on the metabolites eluted during this period, a specific set of MRM transitions was monitored during each period [70].

### 4.7. Statistical Analysis

All statistical analyses were performed using SPSS 26.0 software (SPSS, Chicago, IL, USA). One-way analysis of variance (ANOVA) was used to test the hypotheses, and a difference was considered significant if the *p* value was < 0.05. All quantitative data are expressed as the means ± standard deviations (SDs) of three replicates.

Metabolites from 9 samples (UV-B 0, 10, and 20 d, three replicates for each treatment) were selected for PCA, HCA, OPLS-DA, and Venn analysis using the R package Metabo-AnalystR (http://www.metaboanalyst.ca/ (accessed on 7 March 2022)) to analyze the multivariate and differential accumulation. The data were log transformed (log2) and mean centered before OPLS-DA. Significantly differentially regulated metabolites between the groups were determined based on the following criteria: variable importance in the project (VIP) > 1 and absolute Log2FC (fold change) > 1. The Kyoto Encyclopedia of Genes and Genomes (KEGG) database (http://www.kegg.jp/kegg/compound/ (accessed on 16 April 2022)) was used to annotate the differential metabolites (DMs) and analyze their metabolic pathways. All data were graphed using GraphPad Prism v8.4.3 (GraphPad Software Inc., La Jolla, CA, USA).

## 5. Conclusions

Overall, the present study indicated that although the morphological indicators of *E. brevicornu* leaves only show significant changes after 15 d of UV-B (60 μw·cm^−2^) irradiation, the medicinal components such as icariside I, wushanicaritin, icaritin, and kumatakenin have actually significantly increased after 10 d of irradiation. Accordingly, we suggest that the optimal UV-B irradiation period for *E. brevicornu* is 10–15 days, which can significantly increase their medicinal ingredient content.

## Figures and Tables

**Table 1:** Changes in the microstructure of *E. brevicornu* under UV-B radiation for different days (μm).

Days	Upper Epidermis	Palisade Cell	Spongy Mesophyll	Lower Epidermis	Palisade/Spongy
UV-B 0	13.26 ± 7.58	35.00 ± 8.85	61.89 ± 2.97	9.15 ± 1.16	595.60 ± 97.67
UV-B 5	10.83 ± 0.75	38.62 ± 4.26	70.86 ± 13.34	8.83 ± 0.41	590.21 ± 127.77
UV-B 10	8.78 ± 1.02	32.36 ± 10.34	69.06 ± 5.68	14.03 ± 5.06	490.99 ± 178.91
UV-B 15	8.44 ± 0.64	26.33 ± 1.16	74.64 ± 10.15	8.38 ± 1.19	381.08 ± 55.59 *
UV-B 20	7.83 ± 1.38 *	22.83 ± 3.05 *	70.42 ± 8.13	10.33 ± 6.91	335.46 ± 76.10 *

* *p* < 0.05.

**Table 2:** Significantly increased flavonols.

Compounds	Peak Area (×10^4^)			Log2FC	Log2FC
	UV-B 0	UV-B 10	UV-B 20	(UV-B 0 vs. UV-B 10)	(UV-B 0 vs. UV-B 20)
Kumatakenin	1.93 ± 0.28	6.16 ± 2.69	9.40 ± 1.92	1.67	2.28
Icaritin	863.34 ± 16.30	1871.60 ± 314.28	2127.57 ± 588.18	1.12	1.30
Wushanicaritin	6.69 ± 0.34	22.36 ± 7.26	32.08 ± 19.82	1.74	2.26
Icariside I	1347.30 ± 9.85	4693.27 ± 787.74	4084.00 ± 995.63	1.80	1.60
Kaempferol-3-O-glucuronide	102.46 ± 9.53	222.24 ± 35.19	400.10 ± 136.05	1.12	1.97

**Table 3:** Significantly decreased flavonols.

Compounds	Peak Area (×10^4^)			Log2FC	Log2FC
	UV-B 0	UV-B 10	UV-B 20	(UV-B 0 vs. UV-B 10)	(UV-B 0 vs. UV-B 20)
Baohuoside II	1845.13 ± 24.92	293.34 ± 77.11	686.60 ± 227.09	−2.65	−1.43
Baohuoside III	226.19 ± 29.43	24.93 ± 4.17	62.69 ± 19.95	−3.18	−1.85
5-O-Methylquercetin	16.79 ± 2.82	5.50 ± 3.06	5.71 ± 1.94	−1.61	−1.55
Ikarisoside F	556.51 ± 35.70	104.07 ± 28.47	228.32 ± 78.01	−2.42	−1.29
Isorhamnetin-3-O-rhamnoside	147.40 ± 11.18	67.56 ± 25.52	70.18 ± 39.71	−1.13	−1.07

## Data Availability

All data associated with the study have been reported in this paper. Requests for additional relevant information will be cordially accepted by the authors.

## Appendix A

**Table A1:** Fitting of the binary regression equation for estimation of the *E. brevicornu* leaf area.

Related Factors	Regression Equation	Multiple Correlation Coefficient	Mean Absolute Deviation
X1, X2	Y = 17.962X1 + 27.679X2 − 653.772	0.986	
X1, X3	Y = 3.921X1 + 0.66X3 − 66.676	0.997	18.942
X1, X4	Y = −9.716X1 + 27.679X4 − 653.772	0.986	
X2, X3	Y = −4.681X2 + 0.795X3 + 58.728	0.997	19.094
X2, X4	Y = 9.716X2 + 17.962X4 − 653.772	0.986	
X3, X4	Y = 0.672X3 + 1.723X4 − 56.964	0.997	16.415
X1, X2, X3	Y = 3.745X1 − 4.256X2 + 0.725X3 − 4.648	0.997	21.586
X2, X3, X4	Y = −8X2 + 0.725X3 + 3.745X4 − 4.648	0.997	21.586

Y is the leaf area; X1 is the leaf length; X2 is the leaf width; X3 is the leaf length + leaf width; X4 is the leaf length × leaf width.

**Table A2:** Standard regression equation for flavonols.

Flavonols	Regression Equation	R^−2^	Linear Range (μg/mL)
Epimedin A	Y = 15.902X − 33.942	0.9992	10–200
Epimedin B	Y = 13.412X − 25.350	0.9995	20–400
Epimedin C	Y = 8.107X + 14.032	0.9999	20–600
Icariin	Y = 12.640X + 38.156	0.9991	10–200
Icariside I	Y = 3.039X − 0.889	0.9998	5–40
Icariside II	Y = 12.270X + 4.448	0.9998	1–16

**Table A3:** Flavonoids detected.

Type	Number	Percentage (%)
Flavonols	117	38.49
Flavones	104	34.21
Flavanones	21	6.91
Isoflavones	17	5.59
Flavonoid carbonoside	12	3.95
Flavanols	12	3.95
Flavanonols	11	3.62
Other Flavonoids	5	1.64
Chalcones	4	1.32
Dihydroisoflavones	1	0.33

**Table A4:** Common DMs and their contents.

Compounds	Classification	UV-B 0	UV-B 10	UV-B 20	Log2FC (UV-B 0 vs. UV-B 10)	Log2FC (UV-B 0 vs. UV-B 20)	Log2FC (UV-B 10 vs. UV-B 20)	K-Means
Baohuoside II	Flavonoids	$1.85\times 10^{7}$	$2.93\times 10^{6}$	$6.87\times 10^{6}$	−2.65	−1.43	1.23	4
Baohuoside III	Flavonoids	$2.26\times 10^{6}$	$2.49\times 10^{5}$	$6.27\times 10^{5}$	−3.18	−1.85	1.33	4
Ikarisoside F	Flavonoids	$5.57\times 10^{6}$	$1.04\times 10^{5}$	$2.28\times 10^{5}$	−2.42	−1.29	1.13	4
7-O-Methylnaringenin	Flavonoids	$4.39\times 10^{4}$	$9.05\times 10^{3}$	$1.83\times 10^{4}$	9.24	$1.10\times 10^{1}$	1.72	4
3,4,5-Trimethoxyphenol	Phenolic acids	$1.25\times 10^{5}$	$2.90\times 10^{5}$	$6.32\times 10^{5}$	1.22	2.34	1.12	1
Dehydrodiconiferyl alcohol	Phenolic acids	$3.95\times 10^{4}$	$1.05\times 10^{5}$	$2.52\times 10^{5}$	1.41	2.67	1.26	1
Epipinoresinol	Lignans	$1.69\times 10^{5}$	$4.52\times 10^{5}$	$1.05\times 10^{6}$	2.91	4.07	1.16	1
3,4-dihydroxy-allylbenzene-3	Others	$5.08\times 10^{5}$	$1.81\times 10^{6}$	$3.79\times 10^{6}$	1.12	2.90	1.06	1
Mannitol	Others	9.00	$1.38\times 10^{4}$	$2.86\times 10^{4}$	$1.06\times 10^{1}$	$1.16\times 10^{1}$	1.06	1
2-O-Di-gallic acyl-glucoside	Tannins	9.00	$5.43\times 10^{3}$	$1.78\times 10^{4}$	$1.08\times 10^{1}$	$1.25\times 10^{1}$	1.66	1
N-(4-O-(Glucosyl)-E-feruloyl)	Alkaloids	9.00	$1.64\times 10^{4}$	$5.19\times 10^{4}$	1.42	2.63	1.22	1
(-)-Jasmonoyl-L-Isoleucine	Organic acids	$1.12\times 10^{5}$	$8.44\times 10^{5}$	$1.89\times 10^{6}$	1.25	2.32	1.06	1
Argininosuccinic acid	Organic acids	$3.83\times 10^{5}$	$9.05\times 10^{4}$	$3.26\times 10^{4}$	−2.08	−3.55	−1.47	4
LysoPC 18:1(2n isomer)	Lipids	$8.73\times 10^{6}$	$1.30\times 10^{6}$	$2.71\times 10^{6}$	−2.74	−1.69	1.05	4
2-α-Linolenoyl-glycerol-1,3-di	Lipids	$5.41\times 10^{5}$	$4.35\times 10^{4}$	$1.02\times 10^{5}$	−3.64	−2.41	1.23	4
Dihydroxyoctadeca-dienoic acid	Lipids	$1.88\times 10^{4}$	$4.49\times 10^{4}$	$9.37\times 10^{4}$	−2.28	−1.27	1.01	1
